# Altered Expression of Human Mitochondrial Branched Chain Aminotransferase in Dementia with Lewy Bodies and Vascular Dementia

**DOI:** 10.1007/s11064-016-1855-7

**Published:** 2016-03-15

**Authors:** Emma L. Ashby, Marta Kierzkowska, Jonathon Hull, Patrick G. Kehoe, Susan M. Hutson, Myra E. Conway

**Affiliations:** 1 Department of Applied Science, University of the West of England, Coldharbour Lane, Bristol, BS16 1QY UK; 2 Dementia Research Group, Faculty of Medicine and Dentistry, University of Bristol, Bristol, BS16 1LE UK; 3 Human Nutrition, Foods, and Exercise, Virginia Tech, 1981 Kraft Drive, 1008 ILSB, Blacksburg, VA 24060 USA

**Keywords:** Branched chain aminotransferase, hBCATm, hBCATc, Glutamate, Alzheimer’s disease, Vascular dementia, Dementia with Lewy body

## Abstract

Cytosolic and mitochondrial human branched chain aminotransferase (hBCATc and hBCATm, respectively) play an integral role in brain glutamate metabolism. Regional increased levels of hBCATc in the CA1 and CA4 region of Alzheimer’s disease (AD) brain together with increased levels of hBCATm in frontal and temporal cortex of AD brains, suggest a role for these proteins in glutamate excitotoxicity. Glutamate toxicity is a key pathogenic feature of several neurological disorders including epilepsy associated dementia, AD, vascular dementia (VaD) and dementia with Lewy bodies (DLB). To further understand if these increases are specific to AD, the expression profiles of hBCATc and hBCATm were examined in other forms of dementia including DLB and VaD. Similar to AD, levels of hBCATm were significantly increased in the frontal and temporal cortex of VaD cases and in frontal cortex of DLB cases compared to controls, however there were no observed differences in hBCATc between groups in these areas. Moreover, multiple forms of hBCATm were observed that were particular to the disease state relative to matched controls. Real-time PCR revealed similar expression of hBCATm mRNA in frontal and temporal cortex for all cohort comparisons, whereas hBCATc mRNA expression was significantly increased in VaD cases compared to controls. Collectively our results suggest that hBCATm protein expression is significantly increased in the brains of DLB and VaD cases, similar to those reported in AD brain. These findings indicate a more global response to altered glutamate metabolism and suggest common metabolic responses that might reflect shared neurodegenerative mechanisms across several forms of dementia.

**Electronic supplementary material:**

The online version of this article (doi:10.1007/s11064-016-1855-7) contains supplementary material, which is available to authorized users.

## Introduction

Alzheimer’s disease (AD) represents 64 % of all dementia cases, followed by dementia with Lewy bodies (DLB) and vascular dementia (VaD) [1]. In addition to specific neuropathology observed post-mortem (such as deposition of amyloid plaques and neurofibrillary tangles), several neurotransmitter profiles are compromised in AD brain, such as the cholinergic system and glutamate [2, 3]. Glutamate is the major excitatory neurotransmitter in the central nervous system (CNS) [4], plays a key role in dendrite/synapse formation and plasticity [5], and is important for learning and memory [6]. Levels of glutamate must be tightly regulated since excitotoxicity causes cellular calcium overload, resulting in neuronal cell death, a pathogenic feature associated with AD [7–10]. Increased glutamate concentration has been reported in temporal and inferior parietal areas in AD, where an inability to remove excess synaptic glutamate is considered to contribute to cell death [11, 12].

Recent work by our group has demonstrated that the human branched-chain aminotransferase (hBCAT) enzymes, which regulate brain glutamate [reviewed in 13], show increased expression in the brains of patients with AD [14]. There are two isoforms, cytosolic (hBCATc, 43,400 Da) and mitochondrial (hBCATm, 41,730 Da) that catalyse the reversible transamination of branched-chain amino acids (BCAAs; leucine, isoleucine and valine) and α-ketoglutarate to form their respective branched-chain α-ketoacids (BCKAs) and glutamate [15, 16]. The cytosolic isoform shows tissue and cell specific expression, primarily restricted to the brain and peripheral nervous system and is found largely in neuronal cells, particularly in the axons and nerve terminals of glutamatergic cells and in the cell bodies of GABAergic neurons [17, 18]. This expression is consistent with the hypothesis that hBCATc contributes to the maintenance of metabolic glutamate pools [19]. In rat retina models, gabapentin, an inhibitor of BCATc, impacted glutamate synthesis by 30 % in isolated rat retina, also supporting a role for BCAT metabolism in brain glutamate metabolism [20, 21]. In AD brain, hBCATc showed regional overexpression (↑28 %), particular to the CA1 and CA4 regions, areas which show pathology at an early stage in AD [14]. This increase could result in increased BCKAs and glutamate indicating a role in neuronal toxicity or could also be a response to toxic insult. Conversely, hBCATm, which is specifically localised to endothelial cells of the vasculature, is proposed to have a potential neuroprotective role by supporting astrocytes through metabolism of excess glutamate generated at the synapse [18]. However, the exact details of this mechanism remain undetermined.

Compromised neurotransmitter profiles are also notable in DLB and VaD. People with DLB exhibit Parkinsonian symptoms which is caused by the accumulation of widespread Lewy bodies (eosinophilic cytoplasmic inclusions with alpha-synuclein as their major component), thus sharing common features with Parkinson’s disease (PD) pathology [22]. However, in PD, Lewy bodies are primarily found in the substantia nigra and locus coeruleus compared to the cortices and brainstem in DLB. Moreover, DLB is often accompanied by varying degrees of AD-related pathology (neuritic plaques and less frequently neurofibrillary tangles), which is sometimes classified as pure DLB (no or minor AD-related changes) and common DLB (marked AD-related pathology) [23]. Although the dopaminergic system is primarily compromised in PD patients, dysregulated glutamatergic transmission has also been reported [24–26]. In a sample set of common DLB patients compared to pure AD cases, the expression of metabotrophic glutamate receptors (mGluR) were significantly decreased and correlated with increased neurofibrillary tangle Braak stage, indicating a shared pathogenesis across DLB and AD [27]. Moreover, experimental models of Parkinsonism have indicated that modulation of mGluR was neuroprotective [28]. These studies indicate that in both DLB and AD, glutamatergic transmission is dysregulated and is likely to contribute to the pathogenesis of these conditions.

The clinical characterisation of VaD is more complex. VaD is a heterogeneous disorder largely resulting from neurodegeneration caused by cerebrovascular disease (e.g. atherosclerosis, small vessel disease, lipohyalinosis and cerebral amyloid angiopathy) with different types of vascular lesions [29, 30]. Cognitive decline can be difficult to distinguish from AD but can result from any of a number of different cerebrovascular events [31]. Yet, the neurochemical profile of VaD shares commonalities with AD such as abnormalities in the cholinergic system. Hypo-perfusion, another contributor to VaD triggers hypoxia and glucose deprivation in nerve cells, resulting in membrane depolarization followed by a release of excess glutamate that causes glutamate toxicity [31]. Overall, while different neurodegenerative mechanisms are involved in AD, DLB and VaD, glutamate toxicity seems to be a common feature that warrants further investigation particularly to gain insights in its regulation.

In this study we first examined the distribution and protein levels of hBCATc and hBCATm in the frontal and temporal cortex in DLB and VaD cases compared to age- and sex-matched controls. We then investigated expression of hBCATc and hBCATm mRNA in this cohort and additionally in a cohort of AD cases to explore whether disease-specific differences in protein levels could be attributable to altered mRNA expression. In this report we highlight significant changes in the levels of hBCATm in the frontal and temporal cortex of VaD cases and in the frontal cortex of DLB cases compared to controls. Not only was hBCATm protein increased multiple forms of the enzyme were also observed—raising the possibility of a neuroprotective role for BCATm or protein modification response to the brain damage that occurs in these diseases. These changes suggest a more global response, perhaps reflecting general neurodegeneration, in response to pathogenic challenges.

## Materials and Methods

### Details of Antibodies and Peptides

Rabbit polyclonal antibodies to hBCATc and hBCATm were purchased from Insight Biotechnology (Wembley, UK) and Abcam (Cambridge, UK). Mouse monoclonal antibody to human glyceraldehyde 3-phosphate dehydrogenase (hGAPDH) was purchased from Santa Cruz Biotechnology (California, USA). Western blot confirmed the specificity of these antibodies; anti-hBCATc and anti-hBCATm antibodies detected overexpressed hBCATc and hBCATm proteins, respectively [18] and anti-hBCATc, anti-hBCATm and anti-hGAPDH antibodies detected hBCATc, hBCATm and hGAPDH in brain tissue homogenates at the expected molecular weights [18]. Previous work has demonstrated the specificity of the hBCATc and hBCATm antibodies for Western blot analysis and immunohistochemistry [18].

### Study Cohort

Brain tissue was obtained from the Human Tissue Authority-licensed South West Dementia Brain Bank, University of Bristol, and the study was conducted with approval by North Somerset and South Bristol Research Ethics Committee. The brains had been divided mid-sagittal at autopsy: the left cerebral hemisphere sliced and frozen at −80 °C for biochemical studies and the right cerebral hemisphere fixed in 10 % buffered formalin for 3 weeks before embedding required cut tissue blocks in paraffin for detailed neuropathological assessment. The cohort studied included AD, DLB, VaD and control brains. The AD group were selected on the basis of diagnosis according to Consortium to Establish a Registry for Alzheimer’s disease (CERAD) criteria of “definite AD” [32] and had a Braak tangle stage of IV–VI. DLB cases had all developed dementia before or within 12 months of the onset of motor symptoms and had multiple alpha-synuclein-positive Lewy bodies in the cerebral cortex and brain stem nuclei, usually including the substantia nigra and the locus coeruleus. Most of these cases also had mild to moderate Alzheimer’s disease-type pathological changes, including Aβ plaques, predominantly diffuse, and neurofibrillary tangles amounting to Braak stages 0–IV. VaD cases had a clinical history of dementia, occasional neuritic plaques (if present), a Braak tangle stage of III or less, histopathological evidence of multiple infarcts/ischemic lesions, moderate to severe atheroma and/or arteriosclerosis, and an absence of histopathological evidence of other disease likely to cause dementia. Control cases had no history of dementia, few or no neuritic plaques, a Braak tangle stage of III or less, and no other neuropathological abnormalities.

For biochemical analysis, we dissected samples of left frontal and temporal cortex (Brodmann areas 9 and 22) from 15 neuropathologically confirmed cases of AD (for mRNA expression studies), 15 of DLB, 15 of VaD and 15 controls (for mRNA and protein studies), with groups matched as closely as possible for age, sex, and post-mortem delay (Table 1). Medical Research Council (MRC) database identifiers, demographic and neuropathological data for this cohort are also summarised in Table 1. For immunoperoxidase staining, a subset of 5 brain tissue samples from the DLB, VaD and control group were selected, (paraffin sections from right frontal and temporal lobe).

**Table 1:** Brain tissue used in the study

MRC database ID	Case	Age (years)	Gender	Post-mortem delay (h)	Braak tangle stage
BBN_9039	DLB1	75	M	53	0
BBN_9064	DLB2	77	M	21	III
BBN_9135	DLB3	97	F	24	II
BBN_9297	DLB4	91	M	25	IV
BBN_9302	DLB5	83	M	28	IV
BBN_9305	DLB6	82	M	52	III–IV
BBN_9316	DLB7	76	F	33	III
BBN_9321	DLB8	86	M	15	III
BBN_9324	DLB9	95	M	21	III–IV
BBN_9334	DLB10	69	M	38	0
BBN_4198	DLB11	85	F	30	IV
BBN_9351	DLB12	79	F	26	II
BBN_4202	DLB13	75	M	69	IV
BBN_9370	DLB14	76	M	26	0
BBN_9384	DLB15	67	F	20	0
BBN_8662	VaD1	81	M	66	0
BBN_8667	VaD2	84	F	20	II
BBN_8669	VaD3	93	M	30	III
BBN_8675	VaD4	83	M	24	II
BBN_8724	VaD5	72	M	41	III
BBN_8799	VaD6	90	F	31	I
BBN_8861	VaD7	89	M	30	III
BBN_8927	VaD8	67	M	54	III
BBN_8944	VaD9	97	F	66	II
BBN_8952	VaD10	84	M	30	II
BBN_8975	VaD11	76	M	40	III
BBN_9108	VaD12	79	M	56	II
BBN_9224	VaD13	89	F	22	I
BBN_4208	VaD14	78	F	54	0
BBN_9387	VaD15	89	M	45	II
BBN_8853	AD1	96	F	53	IV
BBN_8960	AD2	78	M	22	V
BBN_8997	AD3	74	F	12	VI
BBN_9122	AD4	83	F	5	V
BBN_9182	AD5	74	M	24	V
BBN_9280	AD6	76	M	11	V
BBN_9295	AD7	85	M	50	VI
BBN_9304	AD8	61	M	38	V
BBN_9323	AD9	84	F	21	VI
BBN_9338	AD10	95	M	27	IV
BBN_9341	AD11	80	F	51	V
BBN_9343	AD12	80	M	24	IV
BBN_9346	AD13	88	F	64	VI
BBN_4200	AD14	69	M	72	VI
BBN_4204	AD15	65	M	39	V
BBN_8749	C1	88	F	28	II
BBN_8770	C2	89	F	15	II
BBN_8779	C3	69	M	66	II
BBN_8835	C4	73	F	59	I
BBN_8898	C5	83	F	24	II
BBN_8980	C6	72	F	24	0
BBN_8983	C7	78	M	48	I
BBN_9028	C8	76	M	23	II
BBN_9292	C9	73	M	35	III
BBN_9311	C10	93	M	38	III
BBN_9329	C11	80	M	46	0
BBN_9340	C12	94	F	21	II
BBN_9344	C13	92	M	35	II
BBN_9353	C14	94	M	40	II
BBN_9354	C15	85	M	31	II

MRC database identifiers, pathological and demographic data for individual cases

*Key* *AD* Alzheimer’s disease, *C* control, *DLB* dementia with Lewy bodies, *F* female, *M* male, *VaD* vascular dementia

### Immunoperoxidase Staining of hBCATc and hBCATm in Paraffin Sections

Seven µm paraffin-embedded sections of brain tissue from the frontal and temporal lobe were collected on 3-aminopropyl-triethoxy-silane coated slides and placed at 40 °C overnight for drying. Before staining, slides were incubated overnight at 60 °C, dewaxed, hydrated, immersed in methanol containing 3 % hydrogen peroxide to block endogenous peroxidase activity, pre-treated by immersion in boiling in citrate buffer (pH 6) and blocked by immersion in 20 % normal horse serum (Vector Laboratories, Peterborough, UK). Sections were then incubated overnight primary antibody optimally diluted in phosphate buffered saline (PBS) (Insight Biotechnology anti-hBCATc 1:1000, Abcam anti-hBCATc 1:100, Insight Biotechnology and Abcam anti-hBCATm 1:1000, respectively). The following day, bound antibody was detected with biotinylated universal secondary antibody and visualised with avidin–biotin horseradish peroxidase complex (VectaElite ABC complex kit, Vector Laboratories) and 3,3′-diaminobenzidine containing <0.1 % H_2_O_2_ (DAB, Vector Laboratories). Sections were subsequently immersed in 0.16 M copper sulphate to enhance staining and counterstained by immersion in Harris’ haematoxylin. Negative control sections (primary antibody omitted) were included with each immunolabelling procedure. Images were acquired using a Nikon eclipse 50i microscope.

Specificity of anti-hBCATc and anti-hBCATm antibodies (Insight Biotechnology) for hBCATc and hBCATm, respectively, was verified by adsorption experiments with the immunising peptides. Antibodies were optimally diluted in PBS and incubated with a 200-fold molar excess of immunising peptide overnight at 4 °C with agitation. Sections from a control case selected on the basis of having strong hBCATc or hBCATm immunolabelling were incubated in (1) unabsorbed anti-hBCATc/anti-hBCATm antibody (positive control) (2) preabsorbed antibody solution and (3) PBS (negative control). The immunohistochemical procedure was otherwise performed as above.

### Preparation of Brain Tissue Homogenates

Frozen tissue (250 mg) from left midfrontal and temporal lobe was homogenised in 1 mL of 1 % SDS lysis buffer (0.1 mM NaCl, 10 mM Tris pH 6, 1 µM phenylmethylsulfonyl fluoride, 1 µg/mL aprotinin, and 1 % SDS in distilled water) in a Precellys 24 homogeniser (Stretton Scientific, Derbyshire, UK). Total protein concentration for each sample was measured using the Bradford Assay; absorbance values were measured on a FluoStar OPTIMA plate reader (BMG Labtech, Buckinghamshire, UK).

### Measurement of hBCATc and hBCATm Protein by Western Blot

Standard hBCAT proteins and brain tissue homogenates were diluted in 1× NuPAGE^®^ LDS Sample Buffer (Life Technologies, Paisley, UK) containing 5 % β-mercaptoethanol (Sigma-Aldrich, Suffolk, UK) to final concentrations of 10 ng, 30 ng and 20 µg of protein, respectively. Diluted samples were denatured by heating at 95 °C for 5 min and electrophoresed alongside Spectra Multicolour Broad Range Protein Ladder (Fisher Scientific UK Limited, Loughborough, UK) on a NuPAGE^®^ 4–12 % Bis–Tris precast gel (Life Technologies) with NuPAGE^®^ MES SDS Running Buffer (Life Technologies) for 1 h at 185 V. Proteins were subsequently transferred to nitrocellulose membrane (GE Healthcare, Chalfont St Giles, UK) in an X Cell II™ Blot Module (Life Technologies) at 18 V overnight at 10 °C. Protein detection was performed using the SNAP i.d. system (Millipore, Watford, UK) according to the manufacturer’s instructions. Membranes were washed in Tris-buffered saline Tween 20 [TBST (0.05 % Tween 20 in Tris-buffered saline—0.02 M Tris, 0.5 M NaCl, pH 7.5)], assembled in the SNAP i.d. manifold, blocked with 0.5 % non-fat dried milk powder in TBST (centrifuged at 2000*g* for 2 min, 30 mL applied with vacuum), probed with primary antibody diluted in 0.5 % non-fat dried milk powder in TBST (Insight Biotechnology anti-hBCATc 1:1000, Insight Biotechnology anti-hBCATm 1:1000, Santa-Cruz anti-hGAPDH 1:4000) for 10 min, washed in TBST (3 × 10 mL with vacuum), incubated with peroxidase-conjugated secondary antibody (Vector Laboratories) diluted in 0.5 % non-fat dried milk powder in TBST (anti-rabbit 1:5000, anti-mouse 1:5000) for 10 min and washed in TBST (3 × 10 mL with vacuum). All incubations were conducted at room temperature. Membranes were subsequently removed from the SNAP i.d. manifold and immunolabelled proteins visualised using Luminata Forte Western HRP Substrate (Millipore) applied for 2 min, followed by exposure to Amersham Hyperfilm ECL (GE Healthcare, UK) for 1 min and developed. Each membrane was probed twice; first for either hBCATc or hBCATm, and after stripping of the membrane in 0.5 M NaOH (5 min) and washing in TBST (3 × 10 min) for hGAPDH. Films were scanned and band density was measured using NIH Image J Software (developed by Wayne Rasband, National Institute of Mental Health). For each case relative hBCATc and hBCATm expression were adjusted for using hGAPDH density.

### RNA Extraction, Reverse Transcription and cDNA Production

3–5 mm^3^ of frozen tissue from the left midfrontal region and temporal lobe was homogenised in TRIzol reagent (Invitrogen, Carlsbad, CA, USA) in a Precellys 24 homogenizer, incubated for 3 min in chloroform and centrifuged at 12,000*g* for 15 min at 4 °C. The upper aqueous phase was separated and mixed with an equal volume of isopropanol and 30 µg of glycogen (Sigma-Aldrich), incubated for 10 min, and centrifuged at 12,000*g* for 10 min at 4 °C for ribonucleic acid (RNA) precipitation. The RNA pellet was washed with 75 % ethanol, re-suspended in water (Sigma-Aldrich), and treated with DNase-I (40 U, Roche Diagnostics Ltd., West Sussex, UK) to remove genomic DNA. RNA concentration was measured using the Ribogreen RNA Quantification Kit (Invitrogen) and a FluoStar OPTIMA plate reader (BMG Labtech). RNA was reverse transcribed to complementary DNA (cDNA) using the High Capacity cDNA Archive Kit (Applied Biosystems, Foster City, CA, USA): 100 ng of RNA in a total volume of 100 µL was incubated at 25 °C for 10 min, 37 °C for 2 h, followed by inactivation at 85 °C for 5 s. cDNA concentration was determined using the Picogreen DNA quantification kit (Invitrogen) and FluoStar OPTIMA plate reader (BMG Labtech) according to the manufacturer’s instructions.

### Measurement of hBCATc and hBCATm mRNA by Real-Time Polymerase Chain Reaction

Real-time polymerase chain reaction (PCR) was performed using the Applied Biosystems StepOnePlus™ Real-Time PCR system (Life Technologies) with Assay-on-Demand Gene Expression Products (TaqMan^®^ Gene Expression Assay probes, FAM dye-labelled, Life Technologies) for *BCAT1* (BCATc), *BCAT2* (BCATm) and the calibrator gene *GAPDH* (GAPDH). The 20 µL reaction mixture comprised TaqMan^®^ Fast Universal PCR Master Mix (TaqMan^®^, Life Technologies), AOD gene expression assay probe and 10 ng of cDNA. Experiment parameters were set as 95 °C for 20 s followed by 40 cycles of 95 °C for 1 s and 60 °C for 20 s. Each sample was analysed in triplicate and SD > 0.2 were repeated. Relative hBCATc and hBCATm mRNA expression was expressed as the fold difference in hBCATc and hBCATm mRNA of each subject relative to the mean value in control tissue, calibrated in relation to *GAPDH* (constitutively expressed by all cell types)using the 2^−ΔΔCT^ method [33]. The mRNA levels were therefore expressed as exponential functions, 2^−ΔΔCT^ values were used to perform statistical tests and the results were presented as the geometric mean with 95 % confidence intervals for each group.

### Statistical Analysis

Kruskall-Wallis test was used with Dunn’s test for pairwise diagnosis group comparisons of hBCATc and hBCATm mRNA expression. hBCATc and hBCATm protein levels were compared between diagnosis groups by Mann–Whitney *U* test and independent *t* test, respectively. Spearman’s test was used to assess correlations of hBCATc and hBCATm mRNA and protein levels with age and post-mortem delay and Mann–Whitney *U* test was used to assess hBCATc and hBCATm mRNA and protein levels in relation to gender, for each diagnosis group and in groups combined. *p* values <0.05 were considered statistically significant

## Results

### Immunohistochemical Distribution of hBCATc and hBCATm in Frontal and Temporal Neocortex of VaD, DLB and Control Subjects

**Fig. 1:**
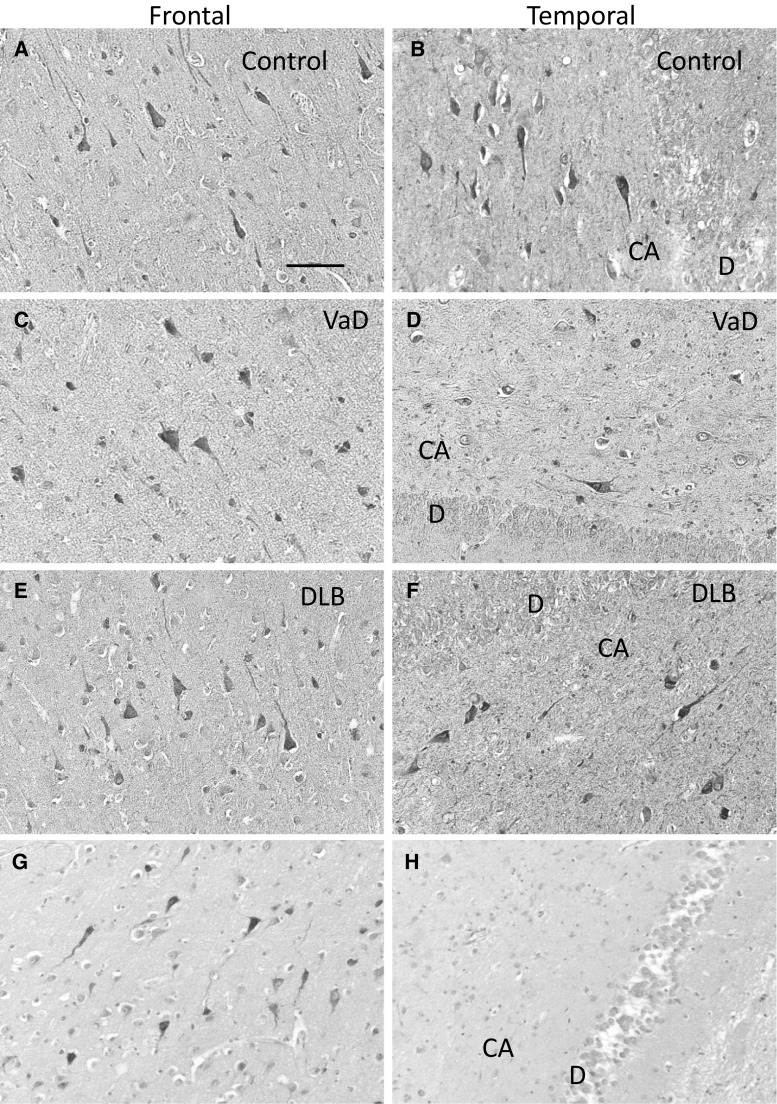
Positive immunostaining for hBCATc in neuronal cells of vascular dementia and dementia with Lewy bodies. Immunolabelling with anti-hBCATc (Insight Biotechnology limited) showed that hBCATc is localised to cortical neurons in frontal and temporal cortex of control (**a**, **b**), vascular dementia (VaD) (**c**, **d**) and dementia with Lewy bodies (DLB) cases (**e**, **f**). Immunolabelling with anti-hBCATc (Abcam) confirmed this neuronal distribution in frontal cortex of the control case (**g**). The specificity of labelling human brain tissue was demonstrated by the lack of signal after pre-incubation of anti-hBCATc (Insight Biotechnologies limited) with 200-fold molar excess of immunising peptide (**i**, control case). The *scale bar* represents 100 µm in **a**–**h**. *DG* dentate gyrus

**Fig. 2:**
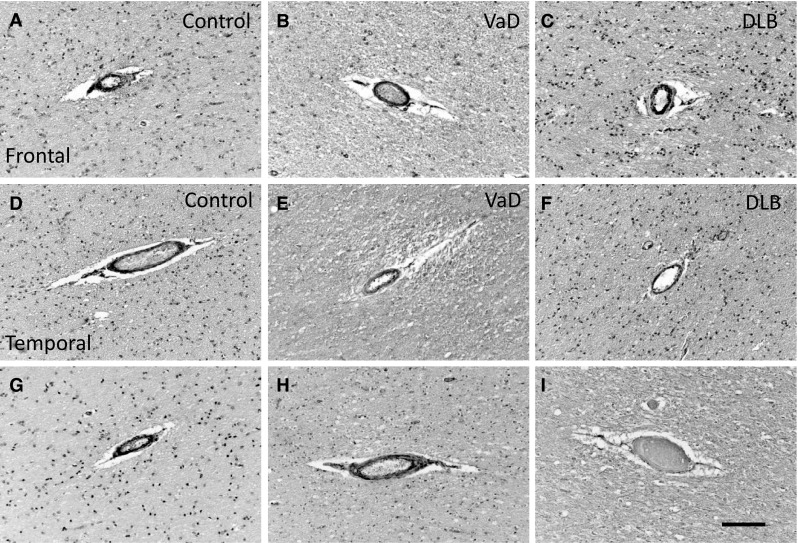
Positive immunostaining for hBCATm in endothelial cells of vascular dementia and dementia with Lewy bodies. Immunolabelling in frontal (**a**–**c**) and temporal cortex (**d**–**f**) with anti-hBCATm (Insight Biotechnology limited) showed that hBCATm is present in the walls of some cortical arterioles in control (**a**, **d**), vascular dementia (VaD) (**b**, **e**) and dementia with Lewy bodies (DLB) cases (**c**, **f**). Immunolabelling with anti-hBCATm (Abcam) confirmed this vascular distribution in frontal (**g**) and temporal cortex (**h**) of a control case. The specificity of labelling of human brain tissue was demonstrated by the lack of signal after preincubation of anti-hBCATm (Insight Biotechnologies limited) with 200-fold molar excess of immunising peptide (**i**, control case). The *scale bar* represents 100 µm

Immunolabelling with two anti-hBCATc antibodies confirmed that hBCATc is distributed in neurons in frontal and temporal cortex (Fig. 1a–g). In all cases there was strong immunolabelling of pyramidal cells in the CA3 and CA4 regions of the hippocampus, whereas neuronal immunolabelling in CA2, CA1 and subiculum was relatively sparse. The specificity of neuronal labelling was demonstrated, where no immunopositive cells were observed following pre-adsorption of the anti-hBCATc antibody (Insight Biotechnology) with 200-fold molar excess of immunising peptide. Immunolabelling with two anti-hBCATm antibodies revealed labelling of the endothelial cell layer of medium sized and large blood vessels, predominantly in white matter, and to a lesser extent in the frontal and temporal cortex (Fig. 2a–h). No other cell types were immunopositive for hBCATm and the specificity of hBCATm immunolabelling was determined by pre-adsorption of the anti-hBCATm antibody (Insight Biotechnology) with 200-fold molar excess of immunising peptide. These findings demonstrate that the cell-specific expression of the hBCAT proteins is similar in all diseased conditions.

### Levels of hBCATc and hBCATm Protein in Frontal and Temporal Neocortex of VaD and DLB Brains Relative to Age- and Gender-Matched Controls

**Fig. 3:**
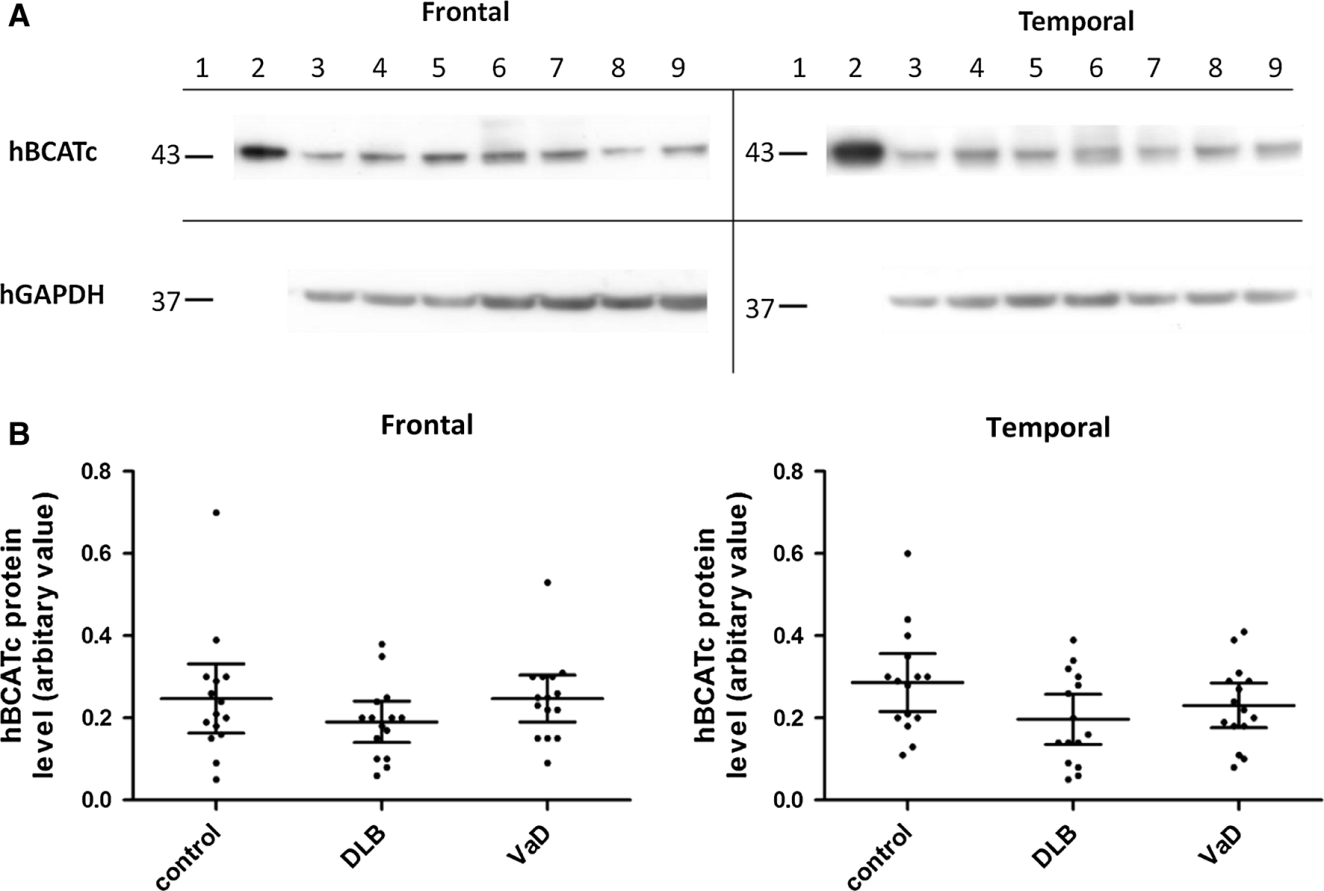
hBCATc protein in frontal and temporal cortex. **a** (frontal and temporal) Representative Western blot probed with anti-hBCATc, followed by anti-GAPDH. For all Western blots, *Lane 1* molecular weight markers, *Lane 2* overexpressed purified hBCATc, *Lanes 3–4* control, *Lanes 5–7* dementia with Lewy bodies (DLB) and *Lanes 8–9* vascular dementia (VaD) homogenates. Anti-hBCATc antibody detected a single band of 43 kDa from overexpressed hBCATc protein and in brain homogenates. **b** Graphs showing hBCATc protein level in frontal and temporal cortex in control, DLB and VaD. The *horizontal bars* indicate the mean and 95 % confidence intervals for each group and each point represents an individual brain

**Fig. 4:**
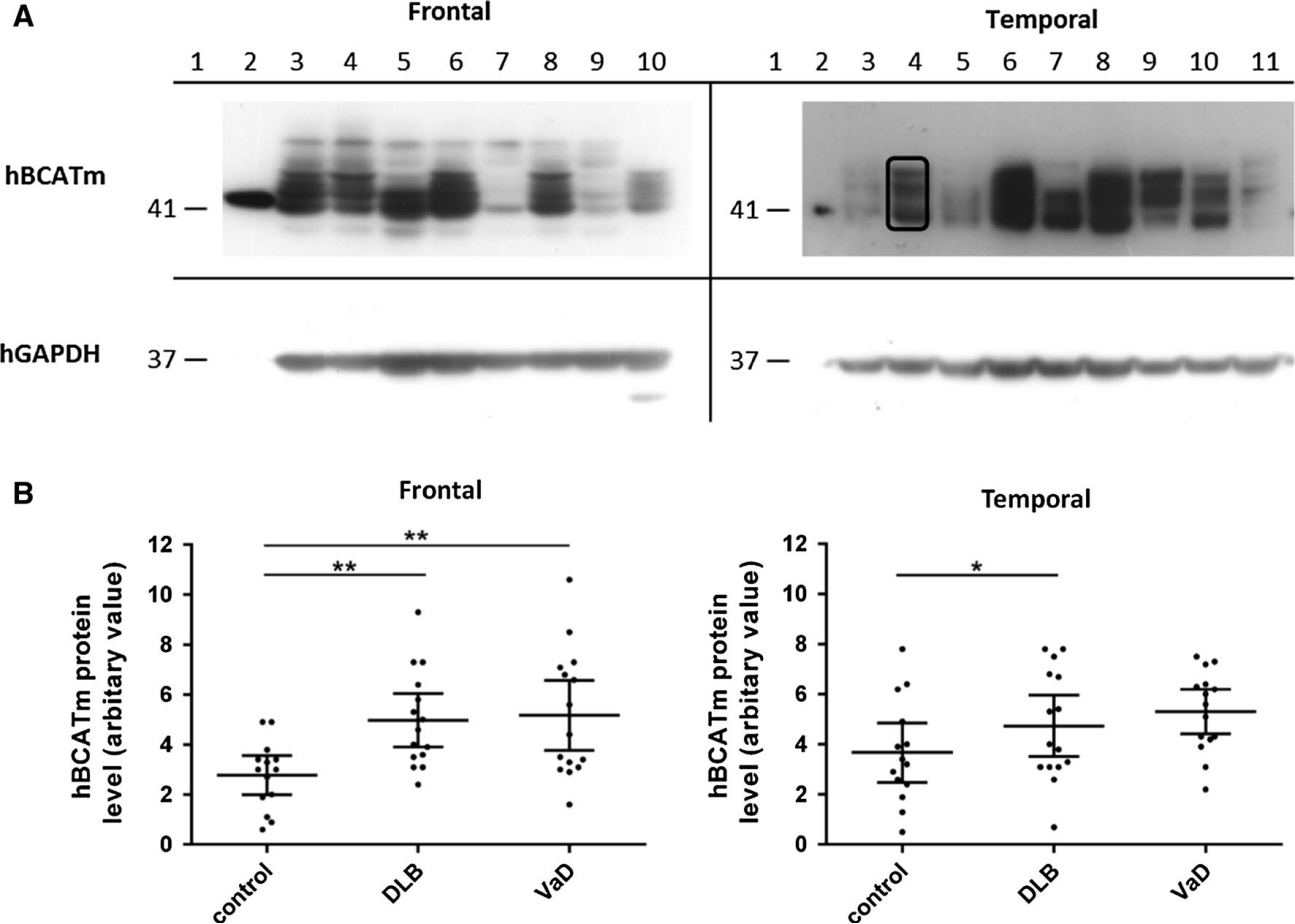
hBCATm protein in frontal and temporal cortex. **a** representative Western blot probed with anti-hBCATm, followed by GAPDH. Densitometry measured the bands specific for hBCATm, as indicated on the blot. For all Western blots, *Lane 1* molecular weight markers and *Lane 2* overexpressed hBCATm, Frontal: *Lanes 3–4* VaD, *Lanes 5–6* DLB and *Lanes 7–9* control homogenates; *Lane 10* AD, Temporal: *Lanes 3, 5, 11* control, *Lanes 4, 6, 7* DLB and *Lanes 8–10* VaD. Anti-hBCATm antibody detected a single band of 41 kDa from overexpressed hBCATm protein and in frontal and temporal homogenates bands between 41 and 52 kDa. **b** Graphs showing hBCATm protein level in frontal and temporal cortex in control, DLB and VaD. The *horizontal bars* indicate the mean and 95 % confidence intervals for each group and each point represents an individual brain

hBCATc and hBCATm protein was measured by Western blot analysis in frontal and temporal cortex of DLB, VaD and control cases. The densitometry from each hBCAT isoform was measured relative to the corresponding GAPDH density in that homogenate (Figs. 3, 4) [14]. After adjustment, the levels of hBCATc protein in frontal and temporal cortex were similar in VaD and control groups and were lower in DLB cases, but this did not reach significance (Fig. 3b).

In contrast, adjusted hBCATm protein differed significantly between VaD, DLB and control brains (Fig. 4b). Adjusted hBCATm protein was significantly increased by 63 % in frontal and 20 % in temporal cortex of DLB cases compared with the control group (frontal: *p* = 0.004, temporal: *p* = 0.024) and were increased by 70 % in frontal and 35 % in temporal cortex of VaD cases compared to controls (frontal: *p* = 0.002, temporal *p* = 0.191).

### hBCATc and hBCATm mRNA Level in Frontal and Temporal Neocortex

**Fig. 5:**
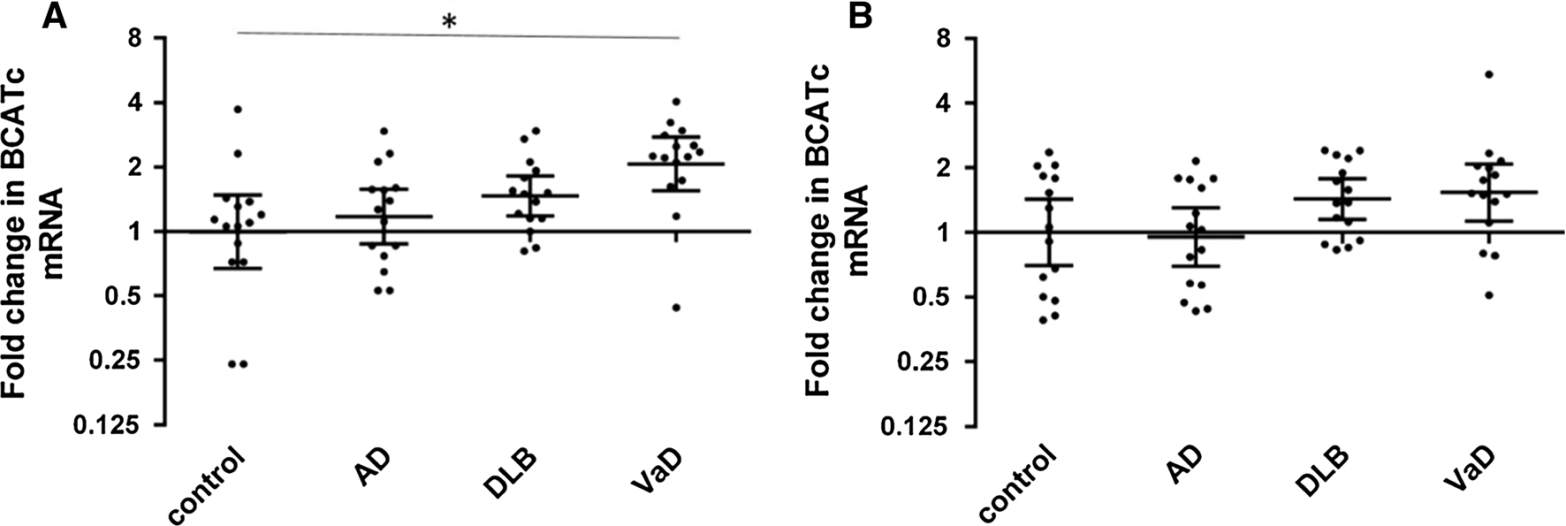
hBCATc mRNA in the frontal and temporal cortex. hBCATc mRNA was measured by real-time PCR in the frontal (**a**) and temporal cortex (**b**) of control, Alzheimer’s disease (AD), dementia with Lewy bodies (DLB) and vascular dementia (VaD) cases. Fold change in *BCAT1* expression in relation to *GAPDH* was calculated using the 2^−ΔΔCt^ method. Graphs show individual data points along with geometric mean and 95 % confidence intervals for each group on a logarithmic scale to the base 2

*BCAT1* (hBCATc) and *BCAT2* (hBCATm) expression were measured by real-time PCR, along with the calibrator gene *GAPDH* (hGAPDH: constitutively expressed in all cell types and has similar expression between diagnosis groups (see Supplementary Figure 1), therefore widely used as a reference gene). In relation to hGAPDH mRNA, hBCATc mRNA levels were similar in AD and control cases in both frontal and temporal regions and were increased in frontal and temporal cortex of DLB and VaD cases compared to controls; however this increase was only significant between the VaD group and the control group (*p* < 0.05) in the frontal cortex (Fig. 5).

**Fig. 6:**
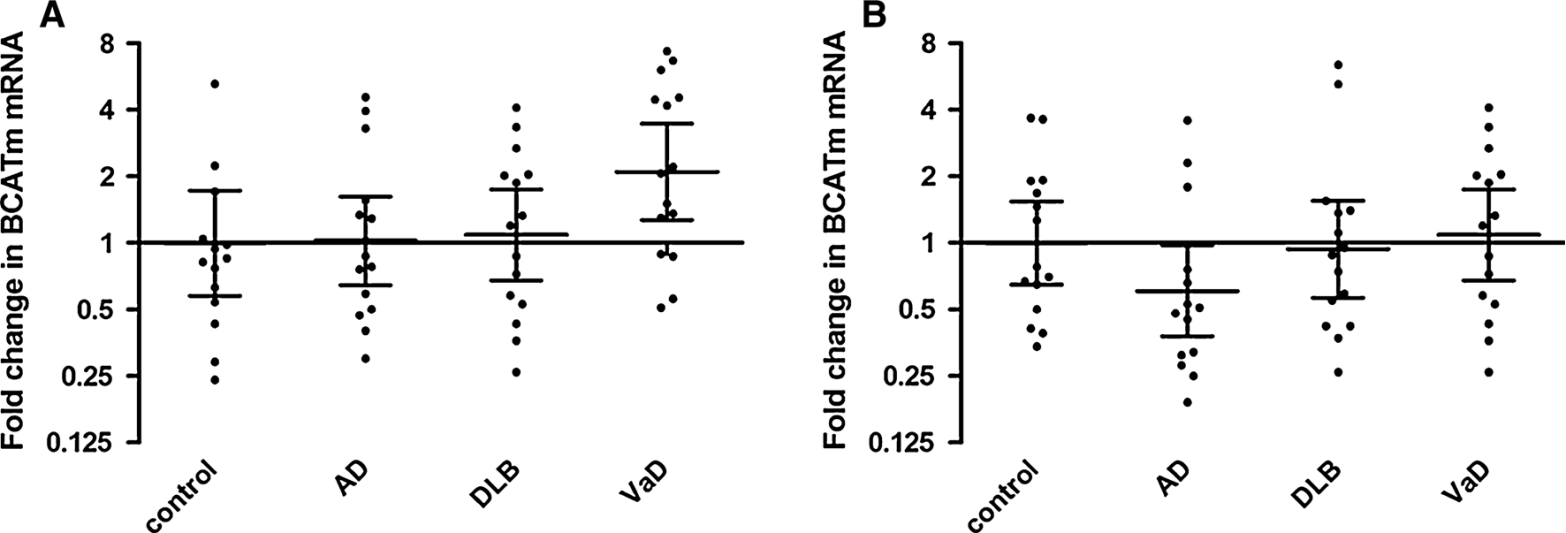
hBCATm mRNA in the frontal and temporal cortex. hBCATm mRNA was measured by real-time PCR in the frontal (**a**) and temporal cortex (**b**) of control, Alzheimer’s disease (AD), dementia with Lewy bodies (DLB) and vascular dementia (VaD) cases. Fold change in *BCAT2* expression in relation to *GAPDH* was calculated using the 2^−ΔΔCt^ method. *Graphs* show individual data points along with the geometric mean and 95 % confidence intervals for each group on a logarithmic scale to the base 2. hBCATm mRNA expression levels were not significantly different between the groups in either of the brain regions

Frontal and temporal hBCATm mRNA levels calibrated in relation to hGAPDH mRNA did not significantly differ between the diagnosis groups, although there was a twofold increase of hBCATm mRNA in the frontal cortex of VaD cases compared to controls and a decrease in hBCATm mRNA in the temporal cortex of AD cases compared to controls, but these did not reach significance (Fig. 6).

### hBCATc and hBCATm in Relation to Gender, Age, Post-mortem Delay and Braak Stage

Protein and mRNA measurements of hBCATc and hBCATm did not vary significantly with gender and showed no correlation with post-mortem delay, as previously reported [28] either in the individual diagnosis groups or with the groups combined. There was a significant positive correlation between hBCATm protein levels and age in the temporal, but not frontal, cortex when diagnosis groups were combined for analysis and in the control group (Table 2) but no correlation with age for any other datasets (hBCATc and hBCATm mRNA or hBCATc protein). What is particularly striking, however, is the presence of multiple bands between 41 and 52 kDa suggesting post-translational modification of the BCATm protein in the vasculature. A significant positive correlation was observed between hBCATm protein expression and Braak stage in both the temporal and frontal cortex (Table 3).

**Table 2:** Statistical analysis of the correlation between hBCATm protein in frontal and temporal cortex with age

Brain region	Analysis of correlation between hBCATm protein level and age by Spearman’s correlation *p* value, r value			
	DLB group (n = 15)	VaD group (n = 15)	Control group (n = 15)	Groups combined (n = 45)
Frontal cortex	0.927, −0.026	0.465, +0.204	0.104, +0.453	0.177, +0.207
Temporal cortex	0.237, +0.325	0.980, +0.007	0.001*, +0.793	0.024*, +0.340

Data are *p* values and r values (Spearman’s correlation) of correlation between hBCATm levels and age in frontal and temporal cortex

*Key* *DLB* dementia with Lewy bodies, *VaD* vascular dementia

* Indicates that correlation is significant at the 0.05 level

**Table 3:** Statistical analysis of the correlation between hBCATm protein in frontal and temporal cortex with Braak stage

Brain region	Analysis of correlation between hBCATm protein level and Braak stage by Spearman’s correlation *p* value, r value			
	DLB group (n = 15)	VaD group (n = 15)	Control group (n = 15)	Groups combined (n = 45)
Frontal cortex	0.235, +0.202	0.492, +0.006	0.409, −0.065	0.047*, +0.259
Temporal cortex	0.016*, +0.555	0.471, +0.021	0.477, −0.016	0.020*, +0.312

Data are *p* values and r values (Spearman’s correlation) of correlation between hBCATm levels and Braak stage in frontal and temporal cortex

*Key DLB* dementia with Lewy bodies, *VaD* vascular dementia

* Indicates that correlation is significant at the 0.05 level

Several steps were taken to ensure the accuracy of data interpretations including assessment of the effects of demographic variables (age, gender and post-mortem delay) on all data sets. Of particular interest from these analyses was the observed positive correlation between age and temporal hBCATm protein expression that was significant when the diagnosis groups were combined for analysis and in the control group. Absence of this correlation in the DLB and VaD groups suggests that the effect of age on hBCATm protein expression is masked by the disease process in which hBCATm expression is elevated.

## Discussion

The incidence of late-onset dementia is predicted to increase three-fold within the next 40 years [1]. This calls for an urgent need for better understanding of the aetiologies of dementias to identify specific drug targets and design effective disease modifying therapeutics. Understanding the profiles and roles of the hBCAT isoenzymes in the CNS may facilitate this, as well as the design of therapeutic compounds that may treat disturbances of the glutamatergic system. In this study we report that the distribution and expression of hBCATc, the neuronal specific protein, is unchanged in DLB and VaD in the frontal and temporal cortex suggesting that in these areas neuronal glutamate metabolism is not affected by BCAA metabolic enzymes. However, Western blot analysis demonstrated a significant increase in the hBCATm protein in the brains of VaD and DLB. Which was also reported in AD brain [14]. Under all disease conditions multiple forms of hBCATm were reported. There was also a twofold increase in hBCATm mRNA in the frontal region of patients with VaD relative to controls, however, this was not observed for the DLB cohort or in the temporal region of VaD, indicating possible alternative mechanisms of hBCATm regulation between conditions and or fundamental differences in the molecular aspects of the disease.

Under normal excitatory conditions a cyclical process exists whereby excess glutamate within the synaptic cleft is taken up by astrocytes and converted to glutamine that is then released to the extracellular fluid for subsequent uptake by pre-synaptic neuronal cells, thereby replenishing neuronal glutamate [34]. However, not all neuronal glutamate is replenished as it is “lost” through oxidation for energy or used as a source of carbon for the production of purines and reducing equivalents such as glutathione [35], but only when glutamate concentrations are high [36, 37]. Here, the BCAAs serve as nitrogen donors to replace this “lost” glutamate and contribute up to 30 % of nitrogen for de novo glutamate synthesis [20, 21, 38–40]. This cycling and regulation maintains levels of neuronal glutamate and prevents toxicity in the synaptic space. However, under conditions where toxicity prevails (such as AD), we have previously demonstrated that hBCATc showed a regional increase in expression in the hippocampus, which could contribute to excess glutamate, described for AD [14]. Increased hBCATm in the endothelial cells of the vasculature was also reported, where a role for hBCATm in neuroprotection was proposed. Here, in this study, we show that the levels of hBCATm are also significantly increased in VaD and DLB, suggesting that the response of this mitochondrial enzyme is a more global reaction, perhaps reflecting a response of the vasculature to general neurodegeneration, in response to pathogenic challenges. We hypothesize that in this instance, if glutamate levels are very high, hBCATm could metabolise glutamate, reflecting the importance of endothelial cells under excitotoxic conditions. Endothelial cells express the excitatory amino acid transporters (EAAT), and can accommodate high concentrations of glutamate, where a concentration gradient in favour of glutamate efflux to blood has been proposed [41]. However, expression of hBCATm in endothelial cells indicates that glutamate can also be metabolised [18]. We have also observed that Glutamate dehydrogenase (GDH) is expressed in these cells (unpublished data), it too can metabolise glutamate generating ammonia and α-keto glutarate. Of course astrocytes are thought to be the main site for glutamate metabolism and uptake, but in conditions such as AD, VaD and DLB, where their role in glutamate clearance is compromised, a support role for the BBB may be important or they may enhance toxicity [42]. Recent studies have indicated that the group I metabotropic receptors (mGluR1 and mGluR5) are altered in post-mortem brains of patients with DLB and may contribute to the AD-like cognitive impairment and plaques found in the DLB brain [27]. The decrease in mGluR1 reported in ‘common’ DLB cases, was suggested to occur potentially due to overstimulation by excess endogenous glutamate, characteristically associated with human and animal models of AD models rather than DLB. As described above for VaD, a reduced uptake of synaptic glutamate could signal increased metabolism by endothelial cells, offering a neuroprotective role through hBCAT metabolism.

Endothelial cells have a high concentration of mitochondria where hBCATm resides [43]. Here, because the concentration of L-glutamate is high, deamination in the direction of α-keto glutarate synthesis is likely, which can feed into the TCA cycle but more importantly reduce toxicity to neuronal cells by removing glutamate. Although evidence in support of auxiliary pathways to remove excess glutamate from brain exist [44–46], the integrity of the BBB is compromised in AD and also in VaD, where increased fragmented vessels, increased capillary basement membrane thickening and reduced microvascular density are all observed [47]. This resonates particularly with VaD as cognitive decline is associated with widespread small vascular lesions, either alone or often coexisting with AD [31]. Part of the neuropathology associated with VaD is significant loss of hippocampal neuronal cells due to impaired microcirculation. Disruptions or damage caused by vascular lesions in this area may cause decreased cerebral blood flow and hypoperfusion contributing to the ischaemia-related damage. Not enough is known about the role of the BBB in some of the less common dementias but altered cerebral blood flow appears to be regional. Therefore the increased hBCATm observed here appears to be a common observation across all of the dementia sub-types, suggesting the potential importance of the vasculature and the BBB in the development of pathology in a number of dementias, especially since hBCATm could serve to lower or exacerbate glutamate concentrations, depending on the direction of transamination.

Not only is the integrity of the BBB negated, as indicated from our studies, we show that multiple bands for hBCATm, not seen for hBCATc, feature in pathological conditions. These higher molecular weight bands may represent different variants of hBCATm or forms of the protein with post-translational modifications, as there was no significant change in mRNA expression. Mitochondria are not only targets but also contribute to cellular stress and in conditions where cellular stress is increased target molecules including lipids, DNA and redox- sensitive proteins are damaged. In response to starvation and increased cellular stress, damaged mitochondria are targeted for degradation, termed mitophagy [48]. Although the underlying mechanisms are not clear current understanding suggests that mitophagy is defective in neurodegenerative conditions, contributing to the accumulation of defective mitochondria [49]. This may in part explain why hBCATm is increased. Moreover, our group previously identified that hBCAT isoenzymes have a well-characterised redox-sensitive CXXC motif located approximately 10 Å from the active site [50] and that hBCAT activity is regulated by the redox potential of the cell in mechanisms involving H_2_O_2_ and NO as oxidising agents [51, 52]. Protein S-glutathionylation is a reversible post-translational modification that can modulate protein activity and has been reported for hBCAT in AD [14]. The process of S-glutathionylation increases globally during overt oxidative stress and locally in the presence of reactive oxygen species [53] and so is likely to be relevant in a number of dementias. Thus, it is possible that post-translational modification of hBCATm could also be causative of the increased levels of hBCATm protein in dementia. This would result in the reversible inactivation of this protein, which may compromise its proposed role in neuroprotection. However, this will depend on the overall exposure to cellular stress, as hBCATm is less easily oxidized. One must also consider that if the function of hBCATm is compromised, GDH metabolism of glutamate could increase levels of ammonia further contributing to toxicity. Thus, increased hBCATm expression may correlate with the underlying vascular pathology of these neurodegenerative conditions and represent a new target to consider when treating human brain excitotoxicity. This concept has been tested in other fields where toxicity-associated damage has been reported. As reviewed by Cederberg et al., [54] ischaemia, subarachnoid haemorrhage, and traumatic brain injury have studied to determine the impact of reducing blood glutamate to alleviate brain toxicity. All studies suggested that by regulating these levels, i.e., lowering glutamate had a positive impact on pathology was reported such as improved recovery, better neuron survival and smaller stroke volume. Moreover, the activity of glutamine synthetase that converts glutamate to glutamine, in the cerebrospinal fluid was also found to be increased not only in AD patients but also VaD and ALS, further indicating that the glutamate/glutamine cycle is compromised in neurodegenerative conditions and that alteration of blood glutamate may be beneficial in treating these toxic episodes [55, 56].

There was no significant increase in hBCATc expression in either the frontal or temporal cortex of DLB, indicating that hBCATc expression is not impacted in these areas. However, our study does not exclude the possibility that regional increases could occur in other brain areas more prone to DLB pathology as has been observed for the hippocampal region in AD. Interestingly, hBCATc mRNA levels were increased in DLB and VaD cases compared to controls and to AD cases, however there were no corresponding increases in hBCATc protein. This could point to some secondary non-specific expression that is less likely to be biologically meaningful in neurons; however it may also point to disease-associated changes in translation or perhaps even changes at a microRNA level.

Collectively, these studies indicate that astrocyte and endothelial glutamate homoeostasis is dysregulated in neurodegenerative conditions where glutamate toxicity features. These studies support a role for hBCATm in protecting via amination of αKG or alternatively enhancing toxicity via leucine transaminaton in endothelial cells. However, the compromised BBB and multi-variant forms of hBCATm observed in these pathogenic conditions may explain why these systems do not operate optimally in neurodegenerative conditions. Future studies clarifying the importance of blood glutamate scavengers may be an important therapeutic target for these hard-to-treat conditions.

## Electronic supplementary material

Below is the link to the electronic supplementary material.

Supplementary material 1 (DOCX 85 kb)
