# Supplementary material for: Altered Expression of Human Mitochondrial Branched Chain Aminotransferase in Dementia with Lewy Bodies and Vascular Dementia
Source: Neurochem Res. 2016 Mar 15;42(1):306–19. doi: 10.1007/s11064-016-1855-7 (PMC5283609; doi:10.1007/s11064-016-1855-7)
Supplement: Supplementary file 1 — Supplementary material 1 (DOCX 85 kb) [file 11064_2016_1855_MOESM1_ESM.docx]

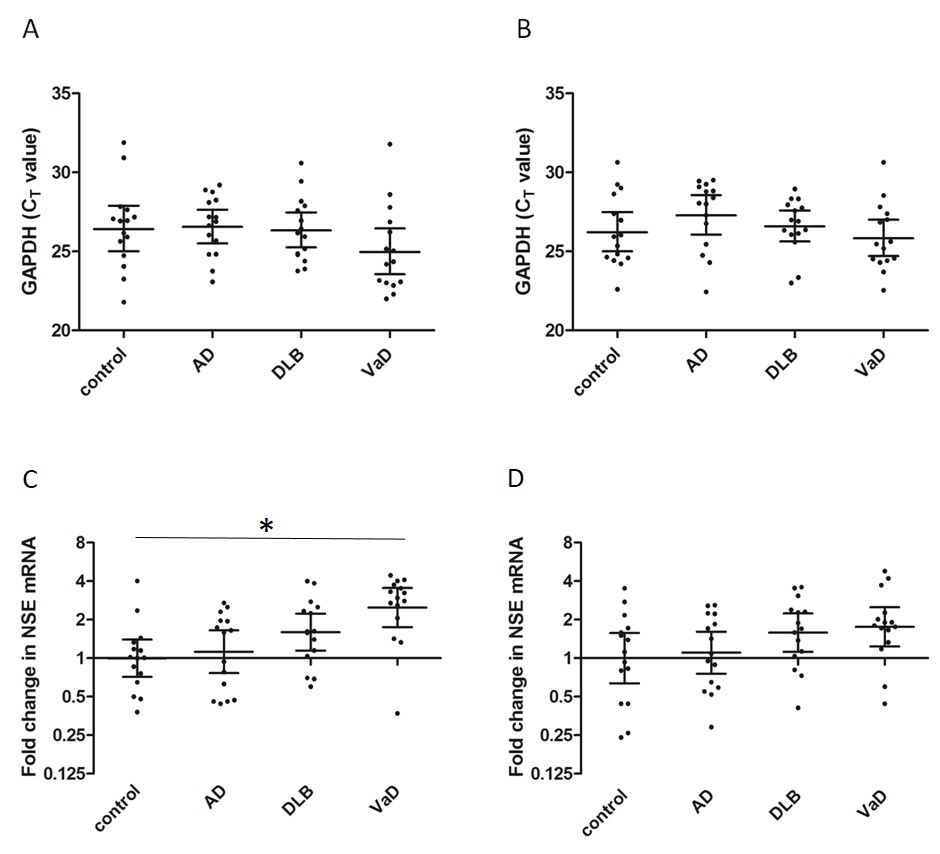


**Supplementary Figure 1 GAPDH mRNA levels measured by real-time PCR in frontal and temporal cortex.** GAPDH transcripts were measured by real-time PCR in frontal (**A**) and temporal cortex (**B**). Interval plots show the mean and 95% confidence intervals of the average C_T_ values in AD, DLB, VaD and control groups and average C_T_ values for each case are shown. GAPDH mRNA levels were similar in all cohorts in both frontal and temporal regions.
